# Clinical Efficacy of Acupuncture on Rheumatoid Arthritis and Associated Mechanisms: A Systemic Review

**DOI:** 10.1155/2018/8596918

**Published:** 2018-04-12

**Authors:** Pei-Chi Chou, Heng-Yi Chu

**Affiliations:** ^1^School of Chinese Medicine, College of Chinese Medicine, China Medical University, Taichung, Taiwan; ^2^Department of Rehabilitation, Tri-Service General Hospital, National Defense Medical Center, Taipei, Taiwan

## Abstract

**Objective:**

The objective of this review is to investigate the detailed existing scientific information about the clinical efficacy of acupuncture on rheumatoid arthritis (RA) conditions and to reveal the proposed mechanisms.

**Methods:**

We searched the PubMed, EMBASE, Cochrane, AMED (Allied and Complementary Medicine), NCCAM (The National Center for Complementary and Alternative Medicine), and CNKI (China National Knowledge Infrastructure) databases to identify relevant monographs and related references from 1974 to 2018. Chinese journals and theses/dissertations were hand searched.

**Results:**

43 studies were recruited. Each research was analyzed for study design, subject characteristics, intervention, selected acupoints, assessment parameters, proposed mechanisms, and results/conclusions.

**Conclusions:**

In our review, we concluded that acupuncture alone or combined with other treatment modalities is beneficial to the clinical conditions of RA without adverse effects reported and can improve function and quality of life and is worth trying. Several important possible mechanisms were summarized including anti-inflammatory effect, antioxidative effect, and regulation of immune system function. However, there is still inconsistency regarding the clinical efficacy and lack of well-designed human/animal double-blinded RCTs. Future discussion for further agreement on taking traditional Chinese medicine (TCM) theory into consideration as much as possible is a top priority.

## 1. Introduction

Rheumatoid arthritis (RA) is the most common inflammatory arthritis and has been known as a chronic and autoimmune disease involving inflammatory condition characterized with symmetrical and persistent synovitis and destructive polyarthritis [1]. RA is also associated with morbidity, chronic disability, and poor quality of life and the cost of care is huge [2, 3]. The estimated prevalence of RA is 0.2–1% [4]. As many factors like susceptibility genes, disease-causing immune cells, cytokine, and signal transduction networks are involved in the pathogenesis of RA [5], the treatment of RA has always been a challenge. The mainstream of the management regarding RA is the use of nonsteroid anti-inflammatory drugs, disease-modifying antirheumatic drugs, analgesics, and biological agents [3]. But the concerns may arise when taking accompanying side effects and toxicity into consideration [6]. Given the fact of the expanding awareness of unwanted side effects of pharmaceutical treatment, there has been an increased utilization of acupuncture as a contemporary healthcare option which has been reported as a kind of safe management [7, 8].

According to traditional Chinese medicine (TCM) theory, RA is categorized under the* “Bi”* or impediment disease, which means a group of diseases caused by the invasion of wind, cold, dampness, or heat pathogen on the meridians involving muscles, sinews, bones, and joints, manifested by local pain, soreness, heaviness, or hotness, and even articular swelling, stiffness, and deformities, also referring to arthralgia.

Acupuncture has been regarded as an important part of TCM and has been used for thousands of years to treat various clinical disorders including* “Bi”* or RA like conditions based on TCM theory. There has been a trend to use complementary and alternative medicine (CAM) as 30–60% of rheumatic patients used CAM [9]. In the United States, a small but significant linear increase in the use of acupuncture (from 1.4% in 2007 to 1.5% in 2012 of the US adults) was reported [10]. About 41% of patients with rheumatic diseases sought the help from acupuncture in Israel [11]. In Taiwan, a recent population-based study revealed the high prevalence and specific usage patterns of TCM including acupuncture in the RA patients [12]. 54.6% of the newly RA user of CAM selected acupuncture only in Korea [13].

However, discrepancy exists between previously conducted investigations and reviews regarding clinical efficacy of acupuncture for RA [14, 15]. As early as in 1985, a literature analysis of the efficacy using acupuncture for RA was done by Bhatt-Sanders and no conclusion was drawn [16]. Ernst and Posadzki suggested that the evidence to support the effectiveness of CAM as a treatment option for RA has also been ambiguous [17]. On the other hand, Hughes concluded that acupuncture elicited a range of effects which contributed to improvements in RA patients' quality of life [18].

The actual mechanism by which acupuncture works also remains controversial. Among all the proposed mechanisms, anti-inflammatory effect has been the most often mentioned which was supposed to provide nonanalgesic effects via suppression of inflammatory response, improvement of blood flow, or relaxation of muscle tone, but they are still largely conjectural [19]. Others include regulating plasma adrenocorticotropic hormone, serum cortisol levels, activity of synovial nuclear factor kappa B (NF-*κ*B), and the release of endorphins [20–22].

In this article, we provide a descriptive and critical systemic review of the current researches available on the clinical efficacy of acupuncture for RA conditions. We analyzed the details of each investigation in terms of study designs, subject characteristics, interventions, outcome assessments, and results/conclusions. We wish to reveal the possible underlying mechanisms as well.

## 2. Material and Methods

A comprehensive search of literatures which were published from 1974 to 2018 was undertaken using the following key words: rheumatoid arthritis, rheumatism, rheumatic disease, acupuncture, electroacupuncture, laser acupuncture, traditional Chinese medicine (TCM), complementary and alternative medicine (CAM), moxibustion, therapeutic effect/efficacy, and their synonyms. These terms were used to search the following databases: PubMed, EMBASE, Cochrane, AME (Allied and Complementary Medicine), NCCAM (The National Center for Complementary and Alternative Medicine), and CNKI (China National Knowledge Infrastructure) databases. Additional articles were also identified from the reference list of identified articles. Chinese journals, theses, and dissertations that we thought might be relevant to our study were hand searched. In order to review as many investigations as possible, we reviewed any article in English or Chinese with full-text available including animal studies. There was no limitation regarding the study type. But we did exclude some articles using the following exclusion criteria:

(1) interventions not involving traditional acupuncture needle insertion or electroacupuncture such as gold thread embedding or bee venom acupuncture but we kept the studies using acupoints stimulation with transcutaneous electric nerve stimulation (TENS), laser, or other herbs;

(2) studies not aiming at the effect of acupuncture on RA such as reliability/validity tests for certain questionnaires;

(3) study protocols not involving human or animal subjects such as expert's opinions or pure descriptive literature reviews/systemic reviews.

## 3. Results

149 studies from 1974 to 2018 were analyzed and 43 studies were recruited into the review. As there are not many researches specific for acupuncture and RA, we tried to include as many articles as possible. The study designs, subjects' characteristics and sample size, acupuncture and other intervention types, applied acupuncture points and their meridians, parameters used for comparison, proposed mechanisms, and the results/suggestions/conclusions of the researches were summarized in Table 1 classified in an order of the year when the study was done.

### 3.1. Study Design

Most of the studies (33 out of 43) are randomized controlled trails (RCT) [22, 23, 25–27, 29, 30, 32–35, 37–45, 48–50, 52, 54–57, 59–62, 66]; 4 of them are double-blinded RCT [49, 54, 61, 62]. There are two single case studies [31, 36]. The rest of the analyzed articles are clinical trials without mentioned randomization or controls groups [24, 28, 46, 47, 51, 53, 58, 63–65].

### 3.2. Subjects

There are studies with human RA patients [23, 24, 28–30, 33–37, 42–66] and studies with animal subjects; most of them are rats [22, 26, 31, 32, 38–41] and rabbits [25, 27] with an established RA model group by in vivo injection of adjuvant components.

Liu et al. included 180 human RA subjects with peptic ulcer to investigate the efficacy of ginger-partitioned acupoint stimulation which is known to be the research with most subjects included [37]. 5 studies contained human subjects more than 100 [23, 29, 37, 42, 60]. 2 researches focused on elderly RA patients [24, 50], one focused on 6 female RA patients [46], and 3 researches recruited healthy human subjects for control as well [33, 34, 66].

### 3.3. Language Used for the Studies

There are 18 researches written in English [22, 24, 31, 34, 36–38, 43, 46, 47, 49, 51–54, 61, 62, 64] and the rest of the analyzed researches in Chinese. English abstracts can be found for most of the studies in Chinese.

### 3.4. Acupuncture Points Applied in the Studies

The selection of acupoints has not been very unanimous according to the authors' clinical experience and TCM theory applied. Some investigations used single acupoint [27, 37, 62] while some used more than 10 acupoints [23, 24, 28, 36, 46, 49, 51, 53, 56–58, 63]. ST36 is the most frequently used acupoint and was selected in almost every research, followed by GB34, LI4, BL60, GB39, and so forth. Please refer to Table 1 for extensive details.

### 3.5. If with TCM Syndrome Differentiation of RA

Several studies mentioned the specific inclusion criteria regarding TCM syndrome differentiation [23, 24, 30, 33, 37, 59] including one animal study [27].

### 3.6. Details of Acupuncture Protocols

The details of the acupuncture protocol are summarized in Table 1. The following describes the detailed items summarized in the table:

(1) Intervention type: traditional acupuncture with different manual techniques, laser acupuncture, electroacupuncture, buccal acupuncture, auricular electroacupuncture, warm needling with or without moxibustion, and acupoint stimulation with herbs.

(2) Modalities used for control or combined therapy: oral or injected form of Western medication, reflexology, moxibustion, herb steaming, massage, mud and sauna therapy, sham acupuncture, autogenic training, herb iontophoresis, oral use of herbs, and electromagnetic millimeter wave.

(3) Treatment frequency/duration and follow-up period of each research summarized in detail as shown in Table 1.

Wang et al. used the same acupuncture protocol but compared the treatment efficacy for 3 and 6 courses [53].

### 3.7. Parameters Used for Efficacy Comparison

#### 3.7.1. Primary Outcomes

Most primary outcome assessments are associated with clinical RA symptoms (pain, morning stiffness, and so forth) and RA symptoms related scales such as pain threshold, visual analogue scale (VAS), pain disability index (PDI), TCM symptom scoring, 28 joints activity index (disease activity score, DAS 28), range of motion of the joint (ROM), 10-meter walk test, grip power, American College of Rheumatology 20 (ACR 20, i.e., 20% of clinical improving rate), ACR 50, and ACR 70.

Parameters specifically used for animals include swelling volume of the joints, limb diameter, and number of swollen joints, arthritis index, skin temperature, and weight.

There are many questionnaires applied in each domain specifically for human RA subjects including quality of life such as the rheumatoid arthritis quality of life questionnaire (RAQoL), health assessment questionnaire (HAQ), short form-36 health survey (SF36), the Pittsburgh sleep quality index, depression, anxiety, and stress scale and face scale for mood, and modified health assessment questionnaire (MHAQ).

Table 1 has the details of each assessment tool used in each study.

#### 3.7.2. Secondary Outcomes

Most of the secondary outcome assessment samples are from the blood and tissues like synovium including two major categories: 

*(1) Regarding Anti-Inflammation*. There are serum levels of erythrocyte sedimentation rate (ESR), C-reactive protein (CRP), rheumatoid factor (RF), interleukins (IL-1, IL-2, IL-4, IL-6, IL-8, IL-10, IL-17, and IL-23), vascular endothelial growth factor (VEGF), immunoglobulins (IgE, IgA, and IgM), white blood cell (WBC), platelet, nuclear factor kappa B (NF-*κ*B), tumor necrosis factor alpha (TNF-*α*), intercellular adhesion molecule 1 (ICAM-1), cholecystokinin-8 (CCK-8), endorphin, protein deacetylase (SIRT-1), myeloid differentiation factor 88 (MYD88), toll-like receptor (TLR4), anti-cyclic citrullinated peptide (anti-CCP), and vasoactive intestinal peptide (VIP). 

*(2) Regarding Antioxidation*. There are superoxide dismutase (SOD), catalase, total antioxidant status (TAS), malondialdehyde (MDA), adenosine triphosphate (ATP), ATP synthase subunit, glutathione reductase (GR), and glutathione peroxidase (GPx).

However, pathological changes of animal tissues (synovium, liver) were also collected [22, 26, 38, 40, 41, 43].

#### 3.7.3. Special Imaging Tools

One study employed positron-emission tomography (PET) scan to detect the regional improvement of inflammation [46] and another study used X-ray of hands for before-after treatment comparison [63].

Please refer to Table 1 for more details.

### 3.8. Proposed Mechanisms

Not every investigation proposed the possible mechanisms of how acupuncture works on the RA condition. Proposed mechanisms could be summarized as the following categories.

#### 3.8.1. Anti-Inflammatory Effect

Among all the mechanisms proposed, this is the single theory suggested by most authors [22, 23, 26, 34, 37–39, 41, 43, 44, 46, 50–52, 55, 58, 59].

#### 3.8.2. Regulating Immune Function

Several studies also indicated the mechanisms to result from regulating immune activities [23, 25, 31, 32, 64–66].

#### 3.8.3. Antioxidative Effect

Some authors believed it to be related to the antioxidation [24, 34, 47].

#### 3.8.4. Miscellaneous

Jie et al. have indicated the fact that there is central analgesic effect by increasing *α*-endorphin level in the cerebrospinal fluid [25], while Adly et al. thought the effects to be via inducing electronic excitation [24]. Specific regulation of the Krebs cycle (also known as the tricarboxylic acid cycle) and glycometabolism were also mentioned in the work of Du et al. [27]. Du et al. suggested the role of biological heat production by acupuncture [33]. For most researches in Chinese, the authors selected the treatment protocol and acupoints according to the TCM theory.

### 3.9. Clinical Efficacy

Almost every investigation found that any kind of acupuncture as the main treatment or adjuvant treatment tool could benefit clinical conditions of RA in human or animal subjects except one [62].

There were no adverse effects of acupuncture reported.

## 4. Discussion

RA has been regarded as a chronic inflammatory condition with various clinical manifestations and some of them could cause serious disabilities and handicaps. Clinicians have been working very hard to suspend the devastating disease progression and deal with the symptoms as well as the impaired function and accompanying stress and cost. However, the actual pathogenesis of rheumatoid arthritis remains incompletely understood. Previous researches have shed light into the cellular and molecular mechanisms and from that base modern Western medications have derived [3]. Contemporary use of medication is linked with the concern of adverse effect and may limit the compliance such as the case of methotrexate (MTX) [67].

An estimated 60–90% of arthritis patients are reported to use CAM including acupuncture [68]. There have been several reviews concerning the clinical efficacy of CAM on rheumatic diseases [8, 9, 17, 69–72] but the latest review specifically focused on the efficacy of acupuncture for RA conditions was conducted in 2008 [14, 15].

Seca et al. suggested a protocol for systemic review focused on pain, physical function, and quality of life but will exclude animal studies and has not been completed [73]. To our knowledge, the present review is the most comprehensive one covering studies from 1974–2018 including human and animal studies and with discussion of the details of study designs, interventions, parameters used for comparison, and the possible proposed mechanisms as well as results and conclusions.

When taking study design into consideration, TCM theory was adapted substantially in most of the investigations. TCM represents the most significant component of complementary and alternative medicine [74]. According to the TCM theory, patients who suffer from the same disease may present different TCM syndrome patterns that also correspond to different biological processes and are associated with different related biomarkers [75]. Lu et al. indicated that RA patients may be divided into cold and heat pattern and they have different molecular signature processes and react differently to certain treatment [76], so theoretically RA patients may be treated by acupuncture without unanimous acupoints according to their TCM syndrome differentiation. These facts have led to obstacles when trying to conduct contemporary researches which critics may face in terms of methodology. Double-blinded RCT is thought to be the most optimal study design to establish scientific evidence, but acupoints selection by TCM theory would experience difficulties which may be the reason of the existing discrepancies between studies of human subjects. In addition, it is also difficult to classify animals into appropriate TCM syndrome categories like human. Another question is the standard localization of acupoints in animals and the correlation and difference between different species.

A RA animal model is often established in animal studies by injection of substance such as bovine collagen [26], Freund's adjuvant [32], or ovalbumin and extra freezing process to imitate the cold syndrome as classified in TCM [27], the disease progression may not be the same in real RA patients, and most of the investigations did not have adequate follow-up period till the chronic stage was achieved (e.g., 10 days [32]). As compared to most human studies, there were at least 3–12 weeks of treatment and follow-up with the longest follow-up period of 3 years [45]. This made the conclusions drawn less practical and applicable for RA patients. But Jie et al. used buccal acupuncture and found that central analgesic effect with upregulation of endorphin and CCK-8 in cerebrospinal fluid could be observed with needle retaining for 30 minutes in RA rabbits; this is the shortest observation but with good results [25].

Some authors provided better clinical efficacy using different needling techniques including warm needling [26–28, 33, 41, 55, 63, 65], plus herb steaming [29], needle-sticking method [59], reinforcing-reducing/twirling-reinforcing needling [27], and moxibustion [57, 66]. EA was used in several studies [22, 30–32, 36, 43, 44, 52, 54, 60], and some authors suggested a better effect than traditional acupuncture [43, 44, 52, 54]. Several studies employed sham acupuncture or EA as the control group [22, 38, 49, 54, 61, 62]. Special forms of acupoints stimulation included laser [24, 34], ginger-partitioned therapy [37], and millimeter waves [61].

We found an interesting fact that acupoint ST36 was used in almost every research and followed by GB34 and LI4. According to TCM theory, RA should fit the disease condition called “Bi” or impediment disease, which means any disease pattern that results from blockage of the meridians occurring when wind, cold, and dampness invade the fleshy exterior and the joints, and that manifests in symptoms such as joint pain, sinew and bone pain, and heaviness or numbness of the limbs as stated in Elementary Questions (Su Wen, bi lun). Distinction is made between three pattern types, each of which corresponds to a prevalence of one of those three evils: wind impediment (or moving impediment) characterized by wandering pain and attributed to a prevalence of wind; cold impediment (or painful impediment) characterized by acute pain and attributed to a prevalence of cold; damp impediment (or fixed impediment) characterized by heaviness and attributed to a prevalence of dampness. A fourth type, heat impediment, arises when the three evils transform into heat. The basic philosophy of how all the acupoints were selected derived from the above theory. As a result, the number of acupoints used seems not to affect the clinical efficacy.

Measurements of quality of life domain have gained more interest among RA patients than other disease-related parameters such as inflammatory biomarkers or joint counts [77]. In this review, some studies have adopted related questionnaires [24, 30, 36, 37, 46, 62] and acupuncture was able to improve the quality of life except in one study [62].

Anti-inflammatory effect has been the most well-known mechanism of how acupuncture works for RA as many studies in this review used inflammatory biomarkers for comparison such as ESR, CRP, RF, IL, NF-*κ*B, and TNF-*α*. Most of the studies comparing these biomarkers indicated the anti-inflammatory effect of acupuncture [22–24, 26, 31, 32, 37–39, 41, 43, 44, 50–55, 58, 59, 66]. Wang et al. indicated the reduction of ESR and CRP after acupuncture in RA subjects in their review as well [15]. Han et al. thought that acupuncture can lower TNF-*α* and VEGF in peripheral blood and joint synovia to improve the internal environment which is beneficial for RA [41].

Dong et al. indicated that toll-like receptor (TLR) signaling pathway contributed to the development and progression of RA and acupuncture could reduce the expression of TLR4, thus leading to anti-inflammation [31]. However, some authors did find clinical effect but not via anti-inflammation [46, 49]. Efthimiou and Kukar indicated that even though no clear anti-inflammatory effect has been demonstrated, acupuncture may still be a useful adjuvant for pain [70]. In our review, we think anti-inflammatory effect acts in certain occasion to improve the RA conditions.

Another possible mechanisms could be attributed to the antioxidative effect (such as inducing the increased activities of SOD and catalase in the serum of RA, alleviating oxidative stress and inflammation, and improving antioxidant and energy metabolic status) [33, 34, 47] and triggered release of endorphins [25] and regulation of immune function as IgG, IgA, and IgM decreased, while IgE did not change evidently after acupuncture in 12 RA patients [23, 32, 64–66].

Forestier et al. concluded in 2009 the evidence level of acupuncture for RA is limited to professional agreement with no scientific evidence [78]. Along with other inconclusive information regarding the clinical efficacy [54, 72], well-designed RCTs are warranted [79]. Most animal studies lack the consistency in establishing the RA model such as the standard injected substance, the dosage, the injected site, the duration of observation after injection, the treatment protocol including acupoints selection, animal acupuncture localization standard, TCM syndrome differentiation, the period of follow-up, and assessment parameters. As for human study, the most important thing is to decide if treatment protocol should vary according to TCM differentiation. Although different mechanism leads to different study design in terms of the parameters that will be assessed, efforts should be put on the standardization and it should be thought whether other methods like functional imaging test would be appropriate.

Acupuncture has its root in TCM and traditionally TCM has one distinguished character; that is, it does not completely seek the specific pathogen and pathological changes in a specific organ or individual, but it seeks the disturbances among the self-controlled systems by analyzing all symptoms and signs. The TCM intervention is based on the differentiation of symptoms to clarify what is wrong in the self-controlled system. TCM seeks the therapeutic mechanism from the integrity and balance, in each individual and between the individual and the environment. The therapeutics work by activating and improving system connection and enhancing human resistance. The mechanism in TCM is not like modern medicine that seeks the mechanism from cellular or molecular perspectives [80]. In light of this, to attend simultaneously to a well-designed RCT with every possible variable controlled and TCM theory is extremely difficult. Future agreement on this issue warrants extensive discussion.

Although we have tried our best searching and analyzing all the eligible articles, some earlier works are not available due to language barriers. We were not able to level the quality of each article as there are not many of them. We also exclude some investigations such as ones using venom acupuncture or gold thread embedding as more information about efficacy and mechanism may not be revealed. Some Chinese articles did bring about the treatment philosophy according to TCM, but it was very hard to organize and summarize well.

## 5. Conclusions

In our review, we concluded that acupuncture alone or combined with other treatment modalities is beneficial to the clinical conditions of RA without adverse effects reported and can improve function and quality of life and is worth trying. Several important possible mechanisms were summarized including anti-inflammatory effect, antioxidative effect, and regulation of immune system function. However, there is still inconsistency regarding the clinical efficacy and lack of well-designed human/animal double-blinded RCTs. Future discussion for further agreement on taking TCM theory into consideration as much as possible is a top priority.

## Figures and Tables

**Table 1:** Summary of researches selected and analyzed.

Authors and year	Study design	Subject characteristics	Interventions	Acupuncture points applied	Outcome assessment	Possible mechanisms proposed	Results and conclusions
Meng et al. 2018 [23]	RCT	160 RA patients	Acupuncture (once daily for 4 weeks) + herbs versus medication	ST36, BL18, BL20, BL23, RN6, RN4, ST36, DU14, LI11, SJ4, ST5, LI4, GB34, GB31, ST34, KI6, BL60, SP10, GB33, EX-LE4, EX-LE5, SI11, SI9, LI15, SJ14, BL36, GB30, BL54, GB40, BL62	RA symptoms, ESR, RF, CRP, IL-1, IL-6, TNF-*α*, ICMAM-1	Inhibiting the inflammatory reaction and improving immune function	(1) Acupuncture + herbs had significantly better effect on RA symptoms (2) Significant differences in all parameters between 2 groups

Adly et al. 2017 [24]	Clinical trial	30 elderly RA patients	Laser acupuncture versus reflexology (12 sessions in 4 weeks for both groups)	LR3, ST25, ST36, SI3, SI4, LI4, LI11, SP6, SP9, GB25, GB34, HT7	RAQoL, HAQ, IL-6, MDA, ATP, and ROM at wrist and ankle joints	(1) Anti-inflammatory effect (2) Radiation absorption by the respiratory chain components inducing electronic excitation (3) Antioxidative	Significant improvement in each group but acupuncture seems to be better

Jie et al. 2017 [25]	Animal study (RCT)	60 rabbits	Body versus buccal acupuncture (needling for 15 s then needle retaining for 30 min in buccal group)	ST36, LE5 “Xi” in buccal region	Pain threshold, cholecystokinin-8 (CCK-8), *α*-EP (*α*-endorphin)	Upregulation of *α*-EP and CCK-8 contents in cerebrospinal fluid (central analgesic effect)	(1) The central analgesic effect of buccal acupuncture is better than body-acupuncture (2) Both buccal acupuncture and body-acupuncture can effectively raise the pain threshold in acute arthritis rabbits

Cai et al. 2017 [26]	Animal study (RCT)	50 rats	Warm needle moxibustion (15 min, once daily for 21 days)	ST36, BL23, GB 39	Swelling volume of the affected knee-joint, IL-1*α*, IL-6, and IL-8, expression of SIRT 1 (a class III histone/protein deacetylase) and NF-*κ*B p 65 proteins in the synovial tissue	(1) Downregulating serum inflammatory cytokines and NF-*κ*B p 65 expression (2) Upregulating SIR T1 expression	Warm needle moxibustion can relieve inflammatory reactions of RA rats

Du et al. 2017 [27]	Animal study (RCT)	40 rabbits	Heat-reinforcing needling (HRN) versus reinforcing-reducing needling (RRN), twirling-reinforcing needling (TRN) (30 min, once a day for 7 days)	ST36	Pain threshold, local skin temperature, endogenous metabolites in the serum (*α*-ketoglutaric acid, citric acid, succinic acid, glucose, inositol, d-ribose, and D-mannose)	The specific regulation for the Krebs cycle and glycometabolism	(1) The effect of HRN group was significantly better than RRN and TRN group (2) HRN for RA with cold syndrome is effective

Chi and Hsu 2016 [28]	Clinical trial	42 RA patients	Acupuncture (once daily for 10 days, 8 sessions) + warm needle acupuncture	ST36, SP6, LI4, LR3, GB34, LI11, SJ5, SJ4, EX-UE9, EX-UE4, EX-LE5, EX-L E2, SP9, ST34, BL62, BL60, KI6, KI3, Ashi points	RA symptoms, RF, ESR	TCM theory	Total effective rate was 95.2%

Fong and Chao 2016 [29]	RCT	120 RA patients	Acupuncture (once daily for 15 days, 2 sessions) with herb steaming versus herb steaming only	LI11, LI5, SJ5, LI4, SP10, ST36, GB34, SP9, ST41	RA symptoms	TCM theory	Acupuncture combined with herb steaming had a better effect on RA symptoms than herb steaming alone

Zhou et al. 2016 [30]	RCT	68 RA patients	Electroacupuncture (3 times a week for 12 weeks) + oral medication versus oral medication	BL18, BL 23, GB 39, ST36, LR 3, LI 4	VAS, clinical symptoms, DAS 28, ACR 20, HAQ, TCM symptoms score, ESR, CRP		(1) The effects of the EA + medication group was better than medication group in terms of symptoms and function (2) Adverse reactions can be reduced by EA therapy coordinated with western medicine

Dong et al. 2016 [31]	Animal study (single case study)	1 rat	Electroacupuncture (30 min daily for 28 days)	ST36, BL60	Arthritis index, paw swelling, TLR4, MYD88, NF-*κ*B	Anti-inflammatory by reducing the expression of TLR4, MYD88, and NF-*κ*B	Acupuncture may play an important role in treatment of adjuvant arthritis rat

Zhang et al. 2016 [32]	Animal study (RCT)	32 rats	Electroacupuncture (30 min, once daily for 10 days) versus medication (prednisolone)	ST36, BL60	Rats' left ankle diameter, serum TNF-*α*, IL-1*α*, and ICAM-1	Downregulating the levels of serum TNF-*α*, IL-1*α*, and ICAM-1	(1) EA intervention is effective in relieving RA rats' inflammatory reaction (2) No significant differences between the medication and EA groups

Du et al. 2016 [33]	RCT	60 RA patients + 30 healthy subjects as control	Heat-reinforcing needling (HRN) versus uniform reinforcing-reducing needling (URN) (once daily, 5 days a week, two weeks)	CV4, CV6, ST36	TCM symptom scoring system, the expression of plasma ATP synthase subunit O (Atp5 O) mRNA and lysosomal V 1 subunit B 2 (At p 6 V 1 B 2) mRNA	Upregulating expressions of plasma Atp 5 O mRNA and Atp 6 V 1 B 2 mRNA	Both HRN and URN can improve RA patients' clinical symptoms while HRN was better

Attia et al. 2016 [34]	RCT	30 RA patients and 20 healthy subjects	Laser acupuncture (3 days/week for 4 weeks)	LI4, TE5, LI 11, DU 14, LIV3, SP6, GB34, and ST36	SOD, GR, catalase, GSH, plasma ATP concentration, plasma MDA, serum nitrate and nitrite, serum CRP, plasma IL-6, GPx activity, ESR, DAS28 score	Alleviating oxidative stress and inflammation, improving antioxidant and energy metabolic status	(1) The study group revealed significantly increased plasma SOD, GR, GSH, and plasma ATP concentrations (2) Significantly reduced plasma MDA, serum nitrate and nitrite, CRP, IL-6 GPx activity ESR

Chen 2015 [35]	RCT	78 RA patients	Group A = western medication; B = A + herb; C =A + B + acupuncture (once daily for 7 days, 24 sessions)	ST35, EX-LE2, ST36, SP10, Ahi points	RA symptoms	Not mentioned	Total effect in group C was significantly better than groups A and B

Shetty et al. 2015 [36]	Single case study	1 RA subject	EMMS (electroacupuncture, massage, mud, and sauna therapies) (15–45 min for 3 weeks)	GV20, LI4, LI11, BL11, GB4, SP6, KI3, ST44, EX28, and EX36	VAS, 10-meter walk test, isometric hand-grip test, The Pittsburgh Sleep Quality Index, Depression Anxiety and Stress Scales, SF-36, health survey, blood and urine analysis	Not mentioned	The EMMS therapy might be considered as an effective treatments in reducing pain, depression, anxiety, and stress with improvement in physical functions, quality of sleep, and QOL in patient with RA

Liu et al. 2015 [37]	RCT	180 RA subjects with peptic ulcer	Ginger-partitioned acupoint stimulation (15 min, twice daily for 2 months) versus antirheumatic drugs (ARD) versus combination treatment	ST36	RA symptoms, gripping strength, self-reported pain score, DAS-28 RA disease activity score, HAQ, RF, anticyclic citrullinated peptide (anti-CCP), ESR, and CRP	TCM theory/anti-inflammatory effect	Combination treatment with ginger-partitioned acupoint stimulation, oral sanhuangwuji powder, and ARDs had a better clinical effect for RA with complicated peptic ulcer

Li et al. 2015 [38]	Animal study (RCT)	60 rats	Acupuncture (15 min daily for 3 weeks) versus sham acupuncture	ST36, GB39, BL23	Arthritis index, the expression levels of TNF-*α* and NF-*κ*B (p65) in synovial cells, and the content of serum inflammatory cytokines	Acupuncture mediates the anti-inflammatory NF-kB pathway	(1) Parameters were lower for the acupuncture group than for the model group (2) No statistically significant difference between the model and sham acupuncture group

Guo et al. 2015 [39]	Animal study (RCT)	32 rats	Electroacupuncture (EA; once daily for 5 days and rest for 2 days, 3 sessions) versus prednisolone	ST36, BL60	Rats' ankle diameter, IL-17, and IL-23	Downregulating serum and knee-joint IL-17 and IL-23 levels	(1) EA can reduce inflammatory reaction of the ankle-joint in RA rats (2) No obvious differences were found between the EA and prednisolone groups except IL-17 protein expression level

Zhang et al. 2013 [40]	Animal study (RCT)	40 rats	Fire needling (once every 3 days, 8 times) versus medication (MTX)	ST36, EXB2	Weight, swelling rate of foot, joint pain score and polyarthritis index of rats, pathological change of liver tissue	Not mentioned	The fire needling has significant efficacy for rats with adjuvant arthritis without any damage to the liver and seems to be better than MTX treatment

Han et al. 2012 [41]	Animal study (RCT)	40 rats	Fire-needle acupuncture (once every 3 days, 8 times) versus MTX	ST36, EXB2	Rat's right hind paw swelling volume, serum IL-1 and TNF-alpha, pathological changes of synovium tissue of the right knee-joint	Downregulating serum IL-1 and TNF-alpha contents	(1) No significant differences were found in the swollen paw volumes on day 12 (2) Both groups showed better pathological observation

He et al. 2011 [22]	Animal study (RCT)	75 rats	Electroacupuncture (15 min, once every other day for 15 days) versus sham	ST36, GB39, BL23	Body weight, paw volume, histologic inflammation scoring, VIP	Partially through the induction of VIP expression	EA markedly decreased the paw swelling and the histologic scores of inflammation in the synovial tissue and reduced the body weight loss

Gao 2011 [42]	RCT	114 RA patients	Group A = Western medication, B = A + acupuncture (once daily, 5 times a week for 3 months)	RN6, RN4, ST36, BL18, BL20, BL23	RA symptoms	TCM theory	Group B had much better clinical effect than group A

Ouyang et al. 2011 [43]	RCT	63 RA patients	Electroacupuncture (EA) versus simple needling (SN) once every other day for 10 times, 3 sessions	Acupoints were selected mainly from yang-meridian and local Ashi points (pain-point)	TNF-*α*, VEGF in peripheral blood and joint synovia	Lowering TNF-*α* and VEGF in peripheral blood and joint synovia	(1) EA and SN could both reduce the TNF-*α* and VEGF (2) The lowering of VEGF was more significant in the EA group

Ouyang et al. 2010 [44]	RCT	63 RA subjects	Electroacupuncture (EA) versus simple needling (SN) (once every other day for 20 times, 3 sessions)	GV20, GB20, LI11, TE5, CV4, ST36	IL-1, IL-4, IL-6, and IL-10 in peripheral blood and joint fluid	Decreasing the proinflammatory cytokine of IL-1 and IL-6 and increasing the inhibition cytokine of IL-4 and IL-10	(1) Both groups reduced the interleukins (2) EA group had a greater effect than SN group

Liu 2009 [45]	RCT	57 RA patients	Acupuncture (once daily for 15 days, 2 sessions for consecutive 2-3 years) versus medication	SP6, SP9, ST36	Functional assessment	TCM theory	Acupuncture group had significantly better ADL function (81.5% compared with 50.0%)

Sato et al. 2009 [46]	Clinical trial	6 female RA patients	Acupuncture (10 acupuncture treatments in 2 months)	ST34, ST35, ST36, SP9, SP10, BL39, BL40, BL56, KI10, GB31, and GB34	VAS, ROM, face scale (mood), MHAQ, FDG-PET images, ESR, CRP	Not through reduction of regional inflammation	VAS, ROM, face scale and MHAQ improved in all patients and significantly after acupuncture, but no significant change in ESR, CRP, and PET images

Kim et al. 2009 [47]	Clinical trial	21 RA patients: responders (at least 50% reduction in swollen joint counts) or nonresponders (less than 50% reduction in swollen joint counts)	Acupuncture (14 sessions in 6 weeks)	Not mentioned	TAS in the serum, the SOD, catalase	The increased activities of SOD and catalase in the serum	(1) The responders showed significantly greater changes in the activity of SOD (2) No significant differences in the changes of the catalase activity and TAS between the groups

Chen et al. 2009 [48]	RCT	60 RA patients	Muscular acupuncture (once daily for 3 months) versus medication	L11, SP6, and scarring moxibustion on GV14, ST36	RA symptoms, ESR, RF	Not mentioned, possible anti-inflammatory effect	(1) Both groups were effective but with no significant differences (2) Acupuncture caused less adverse effects

Zanette et al. 2008 [49]	Pilot double-blinded RCT	40 RA patients	Acupuncture (AC) versus sham (control AC) (5–10 treatment sessions, followed up at 1 month)	EX 1, PC6, IG4, EX 28, CV 12, CV 6, ST 36, SP 6, LV 3, UB 20, UB 22, UB 23, GV 4, GV 14, UB 11, UB60	ACR20, DAS, VAS, HAQ, ESR, CRP	Not through anti-inflammatory effect	(1) A trend for better efficacy in the AC group (ACR20) (2) Other variables did not differ in both groups

Pang et al. 2008 [50]	RCT	86 RA patients	Acupuncture (once daily for 20 days, 2 sessions) + medication versus medication	DU14, LI11, LI4, SP6, DU3, BL20, RN4, Ashi points	RA symptoms, ESR, CRP, RF, IgG, IgA, IgM	Anti-inflammatory effect	Medication combined with acupuncture group with better clinical effects in terms of each parameter

Lee et al. 2008 [51]	Pilot clinical trial	25 RA patients	Acupuncture (14 sessions for 6 weeks)	HT8, KI10, ST36, SP3, LR8, LR2, SP2, LR1, SP1, SI5, ST41, GB41, ST43, SI3, BL66, SI2, LU8, KI7, SP3, KI3	ACR 20, 50, and 70, DAS28, swollen joint count, SF-36, ESR	Anti-inflammatory effects	(1) At 6 weeks, 44%, 20%, and 12% of patients achieved ACR 20, 50, and 70 responses, respectively (2) Acupuncture also produced statistically significant improvements in DAS28, pain and global activity, swollen joint count, SF-36, and ESR

Bernateck et al. 2008 [52]	RCT	44 RA patients	Auricular electroacupuncture (EA) versus autogenic training (AT) (once weekly for 6 weeks, follow-up at 3 months)	NA	VAS, DAS28, the use of pain medication, the pain disability index (PDI), the clinical global impression (CGI), and proinflammatory cytokine levels	Anti-inflammatory effect	(1) Both EA and AT resulted in significant short- and long-term effects (2) The treatment effects of auricular EA were more pronounced

Wang et al. 2007 [53]	Clinical trial	47 RA patients	Acupuncture: 3 courses versus 6 courses	ST36, CV8, EXUE9, EXLE5, SI3, SI8, LI3, LI4, LI5, LI10, LI11, TE4, TE5, PC7, LU5, LR2, BL62, KI3, KI6. ST41, GB34, SP10	Morning rigidity, swelling, and pain of joints as well as RF, ESR, CRP	Not mentioned, possible anti-inflammatory effect	6 courses had greater effect on parameters than 2 courses of acupuncture treatment

Tam et al. 2007 [54]	Pilot double-blinded RCT	36 RA patients	Electroacupuncture (EA) versus traditional Chinese acupuncture (TCA) and sham acupuncture (Sham) (20 sessions for 10 weeks)	LI11, TE5, LI4, ST36, GB34, GB39	Pain score, changes in the ACR core disease measures, DAS 28 score, and the number of patients who achieved ACR 20 at week 10, ESR, CRP	Not mentioned, possible anti-inflammatory effect	(1) The number of tender joints was significantly reduced for the EA and TCA groups (2) Physician's global score was significantly reduced for the EA group and patient's global score was significantly reduced for the TCA group

Fan and Xia 2007 [55]	RCT	96 RA patients	Acupuncture (heat electroacupuncture instrument with Chinese herb iontophoresis plus medicine) versus control (medicine only) (followed up at one month)	LI11, GB33, GB34, ST34	RA symptoms, CRP, RF, ESR, WBC, platelet	Anti-inflammatory effect	(1) The effective rate was 79.2% in the treatment group and 52.1% in the control group (2) The decreases of blood CRP, ESR, PLT in the treatment group were more significantly as compared with the control group

Chen and Guo 2006 [56]	RCT	137 patients	Acupuncture (once a day for 10 days, 3 sessions) + moxibustion versus acupuncture	SI4, LI5, LI11, SJ5, LI4, ST36, GB34, Sp6, LR3, GB41, EX-UE9, ST41	RA symptoms	TCM theory	Total effective rate was better in group with acupuncture and moxibustion (88.51% versus 64.0%)

Gao et al. 2006 [57]	RCT	98 RA patients	Acupuncture (once daily for 10 days) and moxibustion versus herbs	LI11, SJ5, EX-UE9, EX-LE4, EX-LE5, BL40, GB34, ST36, SP6, GB40, EX-LE10	RA symptoms	TCM theory	Total effective rate was better in group with acupuncture and moxibustion (94.3% versus 80.0%)

Shuain and Hsu 2006 [58]	Clinical trial	20 RA patients	Acupuncture and herbs	LI11, ST36, ST40, AP6, GB39, GB30, PC7, LU5, LI4, SJ5, ST34, GB33, BL60	RA symptoms and ESR	TCM theory and anti-inflammatory effect	Total effective rate was 95%

He et al. 2006 [59]	RCT	50 RA patients	Needle-sticking method versus routine filiform needle therapy (2 sessions)	Not mentioned	RA symptoms (painful and swollen joints), RF, CRP, ESR	Possible anti-inflammatory effect	Both groups had an apparent therapeutic effect on RA, but needle-sticking method was better in terms of RF and symptoms

Ai et al. 2005 [60]	RCT	146 RA patients at active stage	Electroacupuncture versus medication	LI4, LI11, GB34	RA symptoms	Not mentioned	Effective rate was 79.73% in the treatment group and 51.39% in the control group with a significant difference

Usichenko et al. 2003 [61]	Double-blinded RCT	12 RA patients	Electromagnetic millimeter waves (MW) applied to acupuncture points versus sham versus MW exposure 40 min	Not mentioned	RA symptoms	Not mentioned	Patients from MW group reported significant pain relief and reduced joint stiffness during and after the course of therapy. MV may be an adjuvant therapy for RA

David et al. 1999 [62]	Double-blinded RCT	56 RA patients	Acupuncture versus sham (5 treatments at weekly interval for 5 weeks/2 sessions and one 6-week washout period in between)	LI3	ESR, CRP, VAS, global patient assessment, DAS28, GHQ	Not through anti-inflammatory effect	No significant effect of treatment or period and no significant interaction between treatment and period for any outcome variable

Li et al. 1999 [63]	Clinical trial	55 RA patients	Acupuncture + needle warming by moxibustion (once daily for 2 months)	LU9, P7, H7, SP3, LI3, K3, L3, SI3, SJ3, ST43, GB41, UB65, ST36, GB34. GB39, RN4	RA symptoms, ESR, RF, X-rays of hands	TCM theory	(1) The total effective rate was 9 0.9% (2) No changes found in X-rays

Guan and Zhang 1995 [64]	Clinical trial	12 RA patients	Acupuncture (once daily for 10 days, 1–3 sessions)	Ex17, UB12, UB13, LI4, LU7, REN22, LU6, ST36, ST40, LU5, LU9, REN4, REN17	Serum IgG, IgM, IgA	The reinforcement of the immunological function	IgG, IgA, and IgM decreased, while IgE did not change evidently

Liu et al. 1993 [65]	Clinical trial	54 RA patients	Warm needling versus point injection		NK activity and IL-2	Regulatory effect on the cellular immunological function	The NK activity and IL-2 value in RA patients were found to be lower than those of normal individuals; both increased after treatment

Xiao et al. 1992 [66]	RCT	41 RA patients and 16 healthy subjects as control	Acupuncture with moxibustion versus point injection	Not mentioned	IL-2 levels	An influence on the immunity system through neuroendocrine system to improve the IL-2 production	The IL-2 level in control group was unchanged but increased considerably in two RA groups
